# Chromatin and the Cellular Response to Particle Radiation-Induced Oxidative and Clustered DNA Damage

**DOI:** 10.3389/fcell.2022.910440

**Published:** 2022-07-13

**Authors:** John M. Danforth, Luc Provencher, Aaron A. Goodarzi

**Affiliations:** Robson DNA Science Centre, Departments of Biochemistry and Molecular Biology and Oncology, Charbonneau Cancer Institute, Cumming School of Medicine, University of Calgary, Calgary, AB, Canada

**Keywords:** chromatin, particle radiation, clustered DNA damage, multiply-damaged sites, DNA repair

## Abstract

Exposure to environmental ionizing radiation is prevalent, with greatest lifetime doses typically from high Linear Energy Transfer (high-LET) alpha particles *via* the radioactive decay of radon gas in indoor air. Particle radiation is highly genotoxic, inducing DNA damage including oxidative base lesions and DNA double strand breaks. Due to the ionization density of high-LET radiation, the consequent damage is highly clustered wherein ≥2 distinct DNA lesions occur within 1–2 helical turns of one another. These multiply-damaged sites are difficult for eukaryotic cells to resolve either quickly or accurately, resulting in the persistence of DNA damage and/or the accumulation of mutations at a greater rate per absorbed dose, relative to lower LET radiation types. The proximity of the same and different types of DNA lesions to one another is challenging for DNA repair processes, with diverse pathways often confounding or interplaying with one another in complex ways. In this context, understanding the state of the higher order chromatin compaction and arrangements is essential, as it influences the density of damage produced by high-LET radiation and regulates the recruitment and activity of DNA repair factors. This review will summarize the latest research exploring the processes by which clustered DNA damage sites are induced, detected, and repaired in the context of chromatin.

## 1 Introduction

### 1.1 Sources of Ionizing Radiation Exposure

Human exposure to ionizing radiation (IR) is prevalent, with 85% coming from natural sources. For a typical person, approximately 40%–45% of lifetime IR exposure is incurred from the inhalation of radioactive radon gas and its alpha-particle emitting progeny, which emanates from the earth and is often concentrated within the built environment to high levels (Darby et al., 2005; Yoon et al., 2016; Stanley et al., 2019; Khan et al., 2021; Simms et al., 2021). Exposure to radon can vary widely on an individual level, and depends on how radon levels are shaped by the features of a specific building, human behaviour, psychosocial factors, as well as geography (as geology and regional building codes impact exposure) (Gaskin et al., 2018; Stanley et al., 2019; Cholowsky et al., 2021; Khan et al., 2021; Simms et al., 2021). Alpha particles are comprised of two neutrons and two protons, equivalent to a helium nucleus, and are emitted during the decay of radioactive elements (Sgouros 2008). Alpha emitters can be naturally occurring such as uranium (^238^U), thorium (^228^Th, ^230^Th, and ^232^Th), and radium (^223^Ra, ^224^Ra, ^226^Ra), or man-made like americium (^241^Am) or plutonium (^239^Pu) (U.S.NRC 2020; Moniakowska, Block-Łaszewska, and Strumińska-Parulska 2022; IAEA 2010). Radon-222 (^222^Rn) is an alpha particle emitter with a half-life of 3.823 days (Lide et al., 2005; Singh et al.,2011), and is a colorless, odorless, tasteless noble gas that can enter or be actively drawn into buildings *via* the foundation (Yoon et al., 2016; Vogeltanz-Holm and Schwartz 2018). Outside, radon gas disperses quickly, and so outdoor radon levels remain low on the surface of the earth, and it is likely that high concentration radon gas exposure has been negligible in terms of a significant human evolutionary selective pressure compared to resistance to common background terrestrial and solar radiation. Additional exposure to terrestrial radiation comes from naturally occurring radioisotopes, most commonly ^238^U, ^232^Th, potassium-40 (^40^K) and their decay products (Shahbazi-Gahrouei, Gholami, and Setayandeh 2013). External exposure to these sources can be from soil surfaces and building materials like brick, concrete, and gravel, and internal exposure to radioisotopes which can concentrate in foods like mushrooms (Shahbazi-Gahrouei, Gholami, and Setayandeh 2013; Strumińska-Parulska and Falandysz 2020; Joel et al., 2021). Exposure to cosmic rays from space constitutes a smaller fraction of lifetime IR dose exposure of approximately 10%, with higher doses encountered at higher altitudes or during space travel (Grajewski et al., 2011; Berger et al., 2012). Other sources of IR exposure include X-rays and gamma rays, as well as heavy ion beams that are used in the treatment of cancers with high doses for radiotherapy. IR dose exposures are also common during medical imaging procedures such as CT scans (Dauer et al., 2011; Ribeiro et al., 2020).

**FIGURE 1:**
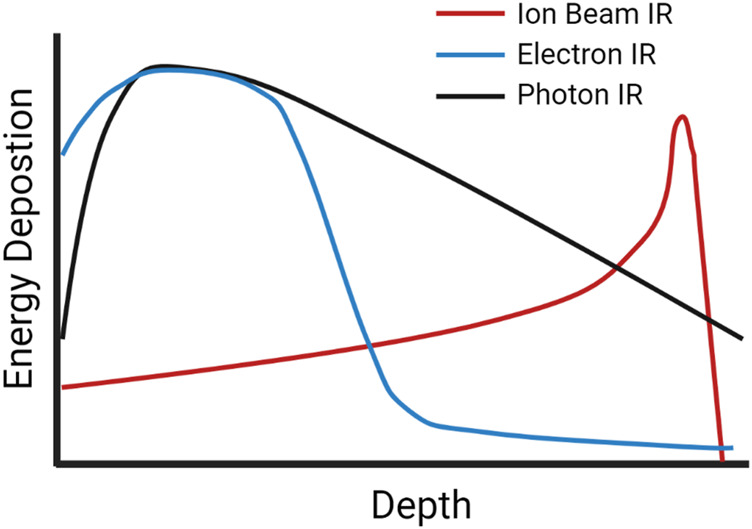
A schematic of dose-depth curves of ion beams and low-LET photon and electron radiation illustrating the difference in peak energy deposition as the radiation travels through tissue.

Photon (X-rays, gamma rays) or particle (alpha particles, beta electrons, neutrons, protons, heavy ions) IR sources can be classified based on LET, a measure of the density of energy deposited along a charged particle as it travels through a medium, for photons this relates to the secondary electrons resulting from photon interactions. The SI units of LET are Joules per meter, J/m, but it is typically expressed in electron volts (eV) per micrometer (keV/µm) (Seltzer et al., 2011). Low-LET X-rays are characterized by a broad energy distribution, and for a treatment beam the peak energy deposition occurs nearer the source and deposition decreases as it progresses through tissue (Song et al., 2012; Desouky, Ding, and Zhou 2015). In contrast, protons, alpha particles and other charged ions have a peak near the end of their pathlength, described as a “Bragg Peak” (Song et al., 2012). Figure 1 shows the dose deposition of low-LET photons and electrons, along with that of ion beams at increasing tissue depth, illustrating the difference in peak energy deposition along an IR track in tissue (Harding, Hill, and Bristow 2013; Kaiser et al., 2019).

Low-LET sources of radiation like X-rays and gamma rays typically have LETs between 0.2–2 keV/μm, high-LET sources such as alpha particles have LETs around 100 keV/μm and charged heavy particles can have LETs as high as several hundred keV/μm. (Park and Kang 2011). Matter exposed to equivalent doses of both IR types will experience a higher density of ionization events after high-LET bombardment, with the energy deposition pattern being dependent on the LET of the IR source. More sparsely ionizing low-LET radiation will produce a more uniform distribution of damage, given the zero mass of photons and their penetrative nature. As photons pass though they interact with the material through a range of process, each leading to the productions of a fast electron (or an electron-positron pair), within the clinic Compton scattering typically dominates with the energy shared between a fast electron and a lower energy photon (Hill 2018). These secondary electrons produce excitation and ionization events, depositing the energy into the surroundings. In the case of fast electrons, sufficient energy can be transferred to other electrons which can then travel significantly beyond its point of origin producing additional excitation and ionization events along its path (Anderson et al., 2017). Conversely, charged ions such as protons and heavier particles will produce damage along the particle track, as well as producing damage *via* secondary electrons, which if they have sufficient energy and travel significantly away from the main track are called delta-rays. For example, low-LET gamma rays exhibit a more dispersed distribution of DNA lesions in a nucleus compared to a greater density of lesions along high-LET tracks observed in cells irradiated with high-LET protons and heavy ions (Cucinotta et al., 2006). Energy deposition patterns are also dependent on the charge and energy per nucleon of the particle, with heavier ions generating greater distributions of high energy delta rays, creating a wider region of damage around the track compared to smaller particles of the same LET (Plante and Cucinotta 2008).

**FIGURE 2:**
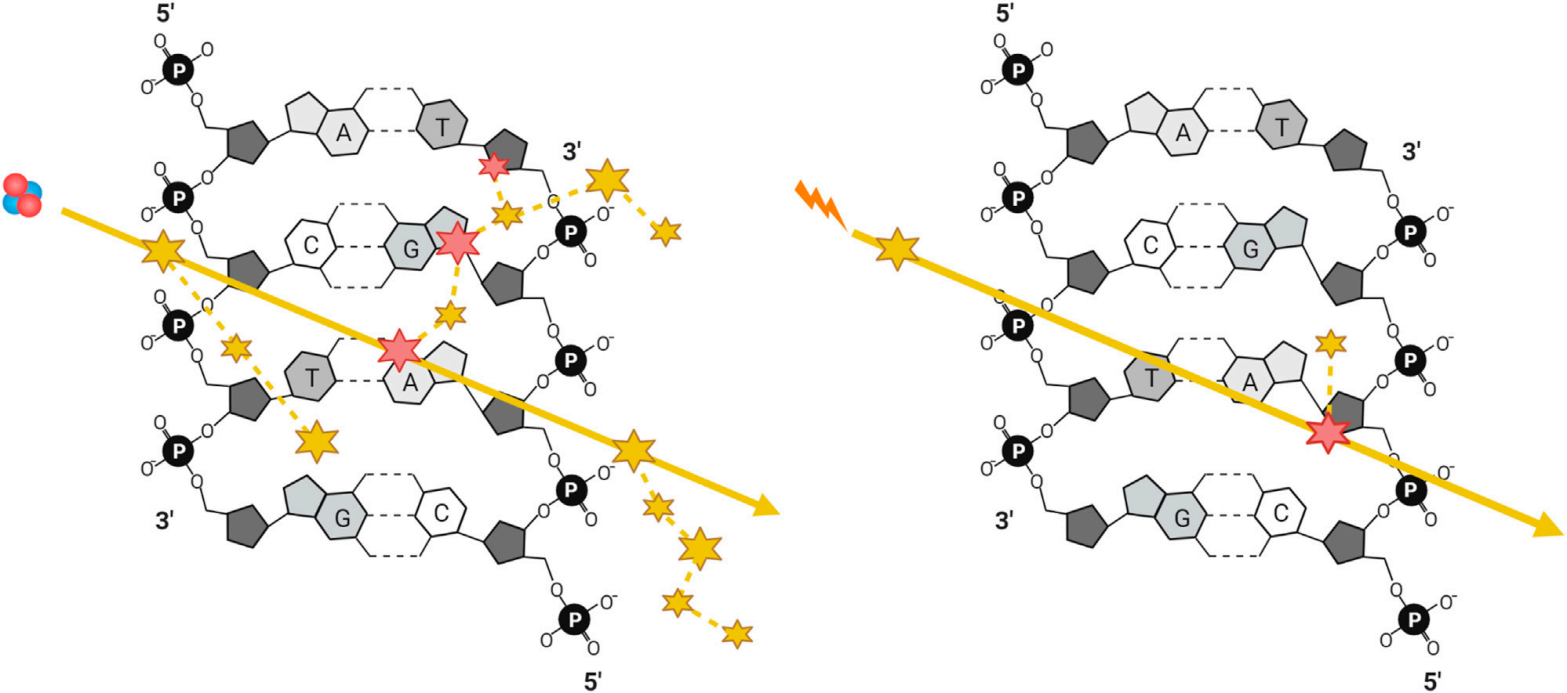
Simplified illustration of energy deposition of equivalent doses of a high-LET particle and a low-LET photon generated secondary electron, with ionizing (large) and excitation (small) events along their trajectories, represented by solid yellow arrows. DNA damage can be generated either directly through ionization (large) or indirectly through generation of radicals after the ionization or excitation of water (small), where dashed yellow lines represent the trajectories of secondary electrons and delta rays.

A comparison of energy deposition of equivalent doses of low- vs. high-LET radiation tracks is visualized in Figure 2 (Park and Kang 2011; Schipler and George 2013). The greater density of energy deposition makes high-LET IR, even at low doses, highly mutagenic due to the challenges presented by the resulting clustered DNA damage. Before the advent of charged heavy ion therapy, much of the work into high-LET IR was done using alpha particles, either from radioisotopes or accelerated ions (Goodhead et al., 1991; Rydberg et al., 2002; Hill 2019). Since the 1990’s a growing body of literature studying the biological effects of high-LET in the context of high doses of carbon ions has emerged, given their use in treating cancers (carbon ion radiotherapy, CIRT). CIRT involves heavy carbon ions produced by particle accelerators such as the Heavy Ion Medical Accelerator in Chiba (HIMAC), Japan (Ebner and Kamada 2016), and is advantageous as it provides greater relative biological effectiveness compared to low-LET IR. Research into the effects of alpha particles in cellular systems has been historically challenging due to the cost and/or logistical difficulties in producing high-LET IR and then accessing them in a routine and/or in a high throughput manner. Although it is true that there have been a number of studies over the past several decades exploring the effects of alpha particles, this body of knowledge is comparatively small relative to the wealth of research performed using low-LET photons. With FDA approval of radium-223 dichloride (Xofigo) for the treatment of prostate cancer in 2013 there has been an increase in the study of the effects of alpha particle damage (Parker et al., 2013). Several alpha particle emitters have been approved for use in radiation therapy, including ^225^Ac, ^213^Bi, ^224^Ra, ^212^Pb, ^227^Th, ^223^Ra, ^211^At, and ^149^Tb [for comprehensive reviews of the clinical advances of targeted alpha particle therapy see (Makvandi et al., 2018; Nelson, Andersson, and Wuest 2021)]. Another commonly employed alpha source is radioactive americium-241, given its high stability with a half-life of 432.6 years and a decay energy similar that to environmental radon-222, it provides a biologically relevant tool for environmental radiation exposure research (Basunia 2006; Singh, Jain, and Tuli 2011).

### 1.2 Ionizing Radiation-Induced DNA Damage

Several forms of DNA damage are induced either directly or indirectly by IR exposure. The terms “damage” and “lesions” will hereafter refer specifically to damage caused to DNA. The most potentially deleterious form of damage induced by IR is the DNA double strand break (DSB) (reviewed in Pearson et al., 2021). For 1 Gy of photon IR exposure, human G_0_/G_1_ phase cells accumulate roughly 20 DSBs, which increases to around 40 DSBs in G_2_ phase as there is twice as much DNA present (Asaithamby and Chen 2011; Bee et al., 2013). DSBs can be formed as a result of two SSBs forming within 1-2 turns of DNA on opposite strands of the double helix. Each SSB may be produced by either a direct interaction *via* the primary radiation or the resulting secondary electrons with the DNA, or indirectly through the excitation or ionization of water resulting in the production of ROS, which diffuse over short distances (<10 nm given the reactive environment of the cell) and react with the DNA (Alizadeh, Orlando, and Sanche 2015; Hill 2018). Thus, DSBs can be generated by two direct, two indirect or a combination of direct and indirect interactions. When direct and indirect interactions occur in isolation, they can produce SSBs and base lesions. Cells maintain redox homeostasis that can be perturbed by a variety of endogenous or exogenous ROS sources including IR (Friedberg et al., 2006). In the case of endogenously generated ROS, there is a considerably lower likelihood that two ∙OH radicals or other damaging agents will occur in close proximity, resulting in a predominant production of DNA single-strand breaks (SSBs) and other single-strand oxidative damage (Friedberg et al., 2006).

**FIGURE 3:**
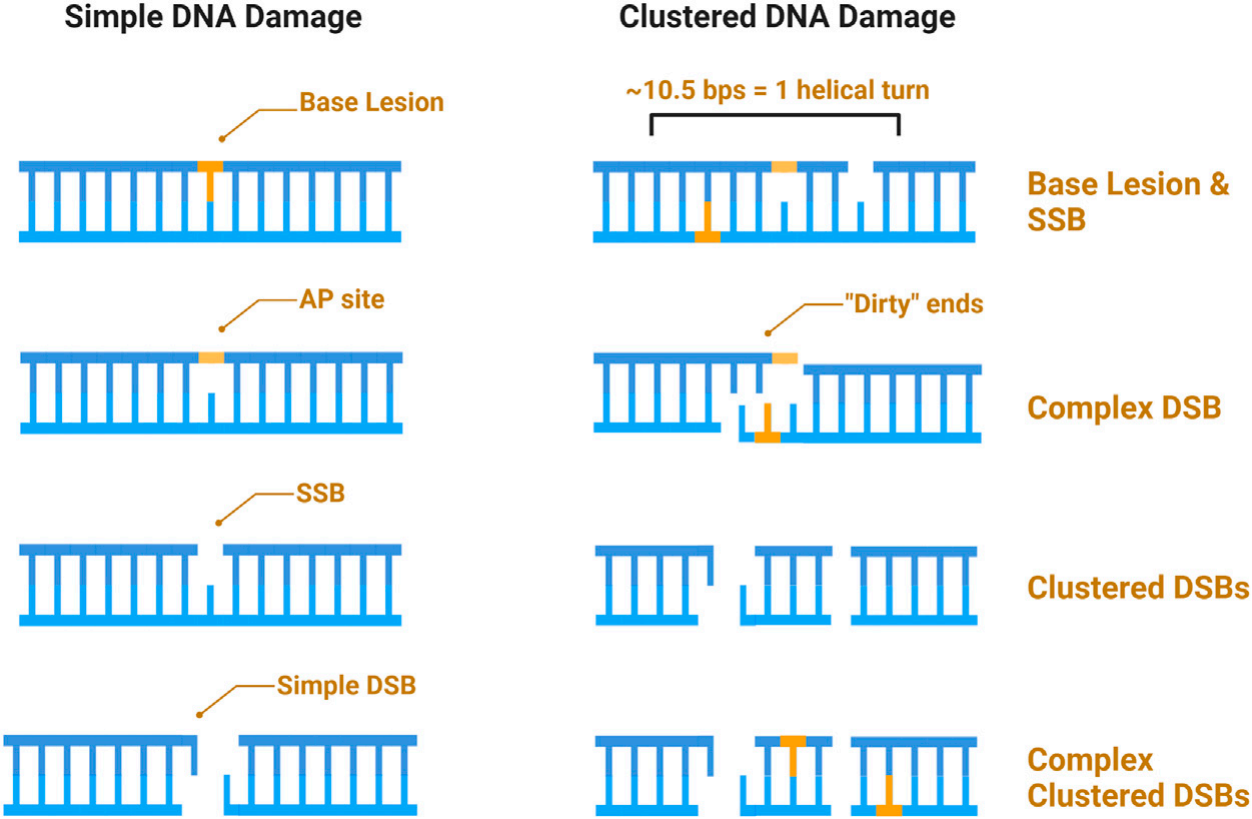
Schematic of IR induced DNA damage. Simple damage includes base lesions such as oxidized bases, apurinic or apyrimidinic sites (AP sites), and isolated strand breaks. Clustered damage occurring within 1–2 helical turns can be comprised of ≥2 simple lesions which may be non-DSB oxidative clustered DNA lesions (OCDLs) or clustered DSBs. Strand breaks can also be complex, with additional base lesions comprising “dirty ends”.

A major characteristic of IR is clustered damage, sometimes referred to as multiply-damaged sites (MDS), and defined as a region wherein ≥2 (of the same or different) lesions occur within 10–15 base pairs, or 1–2 helical turns of each other (Sutherland et al., 2002a; Sutherland et al., 2002b; Sage and Harrison 2011; Mavragani et al., 2017). Due to the density of the ionizing track produced by high-LET IR, this type of radiation is more efficient at generating clustered and complex damage (Eccles, Neill, and Lomax 2011; Lomax, Folkes, and O’Neill 2013). The terms “clustered” and “complex” as descriptors of DNA damage have often been defined vaguely or interchangeably, whereby complex or clustered damage simply refers to multiple types of damage (DSBs, SSBs, base damage) occurring within 1–2 helical turns (Hagiwara et al., 2019). Others have categorized clustered damage into two major categories: clustered DSBs, defined as ≥2 DSBs occurring within 1–2 helical turns of each other, and non-DSB oxidative clustered DNA lesions (OCDLs); throughout this review we will hereafter refer to the former as “clustered damage” and the latter as “OCDLs” (Hada and Georgakilas 2008; Cadet et al., 2012). A caveat to high-LET induced clustered damage is the complexity with which the damage occurs. Here, we define complex damage as the clustering of multiple different types of damage, and so a complex cluster could be a combination of strand breaks or base damage (e.g., a DSB within one helical turn of an SSB or oxidized base). Finally, base damage can occur within a strand break, which we define as a “dirty end.” Examples of each type of defined damage can be found illustrated in Figure 3.

Clustered damage sites were first proposed by Ward in 1981, and subsequent research has found that there is an increase in clusters of complex DSBs after high-LET irradiation (Ward 1981; Hada and Georgakilas 2008). The relationship between the observed amount of clustered DSBs with increasing LET is nearly linear (Nikjoo et al., 2001; Nikitaki et al., 2016), and clustered lesions induced by high-LET IR can contain DSBs that persist >24 h (Riballo et al., 2004; Asaithamby et al., 2008; Stanley et al., 2020). In addition to DSBs, oxidative clustered lesions can be converted into *de novo* DSBs during processing (Cadet et al., 2012; Mavragani et al., 2017), and the proximity of all these lesions to one another proves challenging for DNA repair, leading to the accumulation mutations and chromosomal breaks that increase risk of carcinogenesis (Mavragani et al., 2017).

### 1.3 Impact of Chromatin on DNA Damage and Repair

Our DNA is wrapped around a protein octamer core consisting of two copies of each H2A, H2B, H3, and H4 to form a nucleosome. Though necessary for efficient packing of the genome, this structure can be refractory to cellular processes, especially DNA repair. Base lesions accumulate in a 20 bp range around the nucleosome dyad axis due to glycosylase enzymes’ inefficient activity towards bases in this region (Anderson et al., 2000; Ma et al., 2017; Wu, McKeague, and Sturla 2018). DNA orientation within the nucleosome also regulates glycosylase activity, with more outwardly and accessible bases being more readily removed during repair (Beard, Wilson, and Smerdon 2003; Hinz, Rodriguez, and Smerdon 2010; Rodriguez and Smerdon 2013). These properties of the nucleosome particle in part explain the observation that base lesions accumulate more readily at gene promoters and untranslated regions compared to nucleosome free segments of the genome (Ding, Fleming, and Burrows 2017; Wu, McKeague, and Sturla 2018).

The degree of nucleosome compaction is regulated by epigenetic post-translation modifications on protruding histone N- and C-terminal tails, inducing different signaling events and cellular outcomes. Tightly packed, transcriptionally silent chromatin is referred to as heterochromatin and is characterized by epigenetic markers including elevated H3^K9me3^ and H3^K27me3^, reduced histone acetylation, and increased 5′-methylcytosine (Saksouk, Simboeck, and Déjardin 2015; Allshire and Madhani 2018). Conversely, loosely packed chromatin is referred to as euchromatin, and is characterized with increased histone acetylation (especially at H3^K9^), H3^K4me3^, and reduced DNA methylation (Heintzman et al., 2007; Karmodiya et al., 2012). DNA repair occurs differently in each chromatin environment, and is influenced in terms of pathway choice, speed and mutagenesis due to the unique features of cell transactions specific to those regions (e.g., active transcription, variable chromatin compaction, nucleosome ordering, etc.). In early studies exploring the effect of gamma IR on the induction and repair of DNA lesions in a chromatin context, euchromatic regions were found to be more susceptible to the induction of DSBs compared to heterochromatin, and also exhibited faster repair with distinct genetic dependencies (Falk, Lukášová, and Kozubek 2008; Goodarzi et al., 2008). Heterochromatin was subsequently determined to exert a protective effect on the DNA for low-LET IR (i.e., free radicals), and the frequency of DSBs produced by X-rays in heterochromatin was 5–50 times lower than in decondensed euchromatic regions (Takata et al., 2013). These observations highlighted the important that DNA organization has on the type and amount of damage induced in response to IR, discussed in further detail in the following sections.

## 2 Induction of DNA Damage in the Context of Chromatin

### 2.1 Overview of Commonly Induced DNA Lesions

Base lesions are by far the most common type of DNA damage induced by IR (Sutherland et al., 2000; Pang et al., 2014; Sage and Shikazono 2017). All DNA bases can undergo chemical modifications due to reactions with hydroxyl radicals, leading to a variety of products which can drive mutagenesis. The most prevalent oxidation reactions occur on guanine due to its low redox potential, leading to 8-oxo-7,8-dihydroxyguanine (8-oxoG) and 2,6-diamio-4-hydroxy-5-formaidopyrimidine (FapyG) (Steenken and Jovanovic 1997; Pouget et al., 2002; Douki et al., 2006). Much work has been done with 8-oxoG owing to its ability to pair with adenine, leading to A/T transversion mutations (Shibutani, Takeshita, and Grollman 1991). Adenine can undergo similar reactions to yield 8-oxoA and FapyA, but these are produced in far lower yields than the oxidation products of guanine, likely due to guanine’s propensity to be oxidized compared to other DNA bases. Among the pyrimidines, the most common thymine modifications following low-LET IR are *cis* and *trans* diastereomers of 5,6-dihydroxy-5,6dihydrothymine (known as Thy-Gly). There is a lack of studies examining how cytosine and 5-methylcytosine are modified in cells following IR. For an in-depth review of the oxidation products of DNA, see Cadet and Wagner (2013).

Strand breaks from IR exposure can be generated by direct ionization events or indirectly through the production of ROS *via* water radiolysis. The sparse nature of low-LET radiation results in isolated lesions, and roughly one thousand SSBs generated per 1 Gy of exposure (Sage and Shikazono 2017). The reduced impact of indirect damage in high-LET radiation types, experimentally observed and extensively modelled, suggests that for high-LET IR fewer isolated SSBs are produced compared to low-LET IR, and any clustered SSBs can form nascent DSBs (Wulbrand et al., 2013; Pang et al., 2014; Friedland et al., 2017; Tang et al., 2019; Kundrát et al., 2020; Stanley et al., 2020). DSB induction is observed to be related to LET, with increasing LET producing higher counts of DSBs for equivalent doses (reviewed in (Hada and Georgakilas 2008)). Additionally, with higher LET comes greater complexity, with alpha particles and heavy ions generating far more clustered and complex lesions than equivalent doses from low-LET sources.

### 2.2 Damage Induction by High-LET IR in the Context of Chromatin

Numerous studies have compared the effects of low- versus high-LET IR using comet assays, and demonstrate that high-LET particle IR induces more clustered damage per unit dose compared to low-LET X-rays and gamma rays (Testard and Sabatier 2000; Wada et al., 2002; Rössler et al., 2006). First developed in the 1970s, the comet assay is a microscopy-based method used to quantify DNA strand breaks. Originally carried out under neutral conditions that measures induction of DSBs (Ostling and Johanson 1984), the assay was modified to be carried out under alkaline conditions to monitor single and double stranded breaks as well as alkali-labile sites (Singh et al., 1988). Further enzymatic modifications have been made to the assay to assess the damage from ultraviolet radiation and modified DNA bases (Collins, Duthie, and Dobson 1993; Dušinská and Collins 1996). Detection involved measurement of % of DNA in tail or the tail moment, but there is no consensus as to which of the two measurements is most accurate (Møller 2018).

Combining the neutral comet assay with enzymatic digestion by OGG1, APE1, and NTH (termed enzyme-modified neutral comet assay) can be used to confirm the induction of complex clustered DSBs, which persist for several hours following irradiation with alpha particles (Carter et al., 2018). The same study demonstrated the importance of the E3 ubiquitin ligases RNF20 and MSL2 in the repair of complex clustered DSBs, as siRNA knockdown of these proteins led to persistent comet tails following high-LET IR (Carter et al., 2018). Recently, the enzyme-modified neutral comet assay was used to demonstrate the importance of the deubiquitylating enzyme USP9X in maintaining cell survival following high-LET IR (Nickson et al., 2021). Enzyme digestions can also be employed in pulse field gel electrophoresis experiments. Post-irradiation, *In vitro* digestion with site specific endonucleases such as Nfo (abasic sites), Nth (oxidized pyrimidines), or Fpg as in the comet assays, leads to increased number of DSBs compared to undigested samples, indicative of clustered damage. This methodology was used to demonstrate that the induction of complex damage decreases as chromatin compaction increases with low-LET, with the most damage observed in naked DNA (Magnander et al., 2009).

*In silico* studies have been used to predict the complexity of clustered damage by modeling the energy deposition by various radiation types informed by experimental damage yields. In terms of modeling the chromatin structure within cells, computational models have been developed using input data from studies which used fluorescence *in situ* hybridization and chromatin conformation capture in various cell types to better determine density and distribution of chromatin within a nucleus (Le Dily et al., 2017). Using experimental data for different cell types, the size, geometry, chromatin composition and distribution can be modeled and used to assess the level of damage and quality of the damage induced by simulated IR of various sources.

Early work using open source software was limited by the ability to model SSBs and DSBs induced indirectly by particle IR. The simulation of DNA was constructed in five levels of increasing complexity: 1) DNA double helix, 2) nucleosomes, 3) chromatin fiber, 4) fiber loop, and 5) chromosome territories, and either condensed (structured pattern of fiber loops) or decondensed (randomly distributed nucleosomes) chromatin (Santos et al., 2014). Clustering of damage was defined by 2 or more damage points within 3.2 nm, or approximately 10 bps. From a direct damage standpoint, there was a greater number of small clusters of DSBs (2–3 damage points) in decondensed euchromatin vs. heterochromatin that had a greater proportion of more complex clusters (Santos et al., 2014). After software development needed to simulate indirect damage in the Geant4-DNA platform, modelling was performed for fibroblasts and endothelial cells, finding that a greater amount of DSBs are produced in the euchromatin than heterochromatin for low-LET IR. Heterochromatic DSBs were predominantly produced by directly induced damage, and damage generated in both euchromatin and heterochromatin were of similar complexity (Tang et al., 2019). For simulated alpha IR, higher LET produced a greater proportion of complex DSBs in both euchromatin and heterochromatin.

### 2.3 Chromatin Dependent IRIF Formation

Immunofluorescence microscopy has been used in the identification and quantification of damage foci (Karlsson and Bo 2004; Lassmann et al., 2010; Rothkamm et al., 2015; Sollazzo et al., 2018), and several sub-nuclear complexes that are part of the DSB response have been optimized for imaging DNA repair foci; these are referred to as ionizing radiation induced foci (IRIF) (Goodarzi and Jeggo 2012; Rothkamm et al., 2015). Histone H2AX^S139p^ (γ-H2AX), is a very well-studied marker for DSBs. Once phosphorylated, γ-H2AX signal is amplified along ∼1 Mb of chromatin on either side of the break, and recruits additional signaling, chromatin remodeling and repair proteins (Sedelnikova et al., 2003; Bhogal, Jalali, and Bristow 2009; Plappert-Helbig et al., 2019). After the DSBs are repaired, γ-H2AX is dephosphorylated, and repair capacity and kinetics can be measured by enumerating or quantifying γ-H2AX over time (Chowdhury et al., 2008; Antonelli et al., 2015; Sharma et al., 2015; Ramos et al., 2019; Turner et al., 2019). Widefield epifluorescence microscopy and using z-stacks to acquire 3D images can be used for imaging foci, and immunostaining and visualization of γ-H2AX is sensitive to as little as 1 mGy IR (Bhogal, Jalali, and Bristow 2009; Mah, El-Osta, and Karagiannis 2010). Kinetics of repair using γ-H2AX reveal effective repair of DSBs to baseline levels after exposure to low-LET radiation after 24 h of repair, whereas DSBs induced by high-LET IR show characteristic persistence of DSBs (Asaithamby et al., 2008; Antonelli et al., 2015). Kinetics obtained with by quantifying γ-H2AX by fluorescence microscopy are in agreement with studies using electrophoretic methods (e.g., comet assays) of assessing DNA break formation and repair (Carter et al., 2018; Stanley et al., 2020). All studies show characteristic resolution of DSB damage induced by low-LET X-rays or gamma rays at 24-h post irradiation, which is significantly different from the persistence of DSBs measured by pulsed-field gel electrophoresis (PFGE) and γ-H2AX or strand breaks measured by alkaline comet assays.

As γ-H2AX methods of monitoring DSB repair works by measuring cellular signal transduction events within a chromatin environment (phosphorylation of H2AX histones), it is important to consider several nuances relative to electrophoretic methods that detect physical DSBs. For example, the DSB repair half times measured by PFGE for low-LET X-rays are approximately 20 min faster compared to those measured by γ-H2AX for the same radiation type (Kinner et al., 2008). The apparent lag in kinetics monitored by H2AX methods is partly attributed to the need to signal the presence (and resolution) of DSBs *via* kinase and phosphatase-mediated processes, which require time dictated by enzymatic activities in order to produce changes observable by microscopy. Further, if using signaling events as a proxy for DSB repair, foci number as well as foci size and the relative intensity of signal under the microscope must be considered in order to contextualize the observed kinetics (Löbrich et al., 2010). If γ-H2AX foci size and signal intensity are controlled appropriately during the analysis, then maximum γ-H2AX foci number have been observed as early as 3 min post-IR, which is more comparable to induction times obtained *via* PFGE (Stiff et al., 2004; Kinner et al., 2008; Löbrich et al., 2010). However, due to their small size at very early times, many teams find it more convenient to quantify the induction of γ-H2AX at 15–30 min post IR, a time point long enough for a small portion of fast-repairing DSBs to be resolved, underlying an underestimation (Löbrich et al., 2010). PFGE, on the other hand, may overestimate DSB induction, as the high temperature lysis step can convert heat labile sites into non IR-induced DSBs (Rydberg 2000; Stenerlöw et al., 2003; Kinner et al., 2008; Löbrich et al., 2010).In recent decades the introduction of super-resolution techniques to break through diffraction limited resolution (∼200 nm) barriers have been crucial to delve deeper into the nano-structure of damage repair foci (Natale et al., 2017; Falk and Hausmann 2021; Hausmann et al., 2021). After exposing leukocytes to alpha particles (radium-223) *ex vivo*, single-molecule localization microscopy was used to assess the recruitment of repair proteins to, and the spatial structure of the damage tracks. In single-molecule localization microscopy, fluorescently stained samples are illuminated by standard wide-field light microscopy and the individual fluorescent molecules undergo photo-switching, a process whereby the fluorophore enters its activated “on” state, before inactivating to an “off” or “dark” state a process known as reversible photobleaching. Once these blinking fluorophores are imaged, the individual molecules are computationally reconstructed into an image which can reach resolutions around 20–50 nm (Lelek et al., 2021). As has been found by several studies of γ-H2AX structure using high-resolution techniques, within each large foci there were several nano-foci (Natale et al., 2017; Reindl et al., 2017; Scherthan et al., 2019). Additionally, in some cases larger γ-H2AX foci were observed at the ends of IRIF tracks, these larger “super-foci” were postulated to be a region of higher energy deposition, such as the Bragg peak of the particle track, and found that given the larger size there was a larger number of 53BP1, MRE11, and ATM^S1981p^ (pATM) associated with these super-foci (Scherthan et al., 2019). This was expected and confirms that the size of the γ-H2AX foci is directly proportional to the amount of damage induced.

These γ-H2AX superstructures have also been identified after carbon ion exposure, and visualized with stimulated emission depletion microscopy [reviewed in (Blom and Widengren 2014)] (Reindl et al., 2017). At higher lateral resolution around 105 nm, 53BP1 foci were found to localize at the periphery of γ-H2AX super-structures, showing distinct anti-correlation, and typically found in the peri- and inter-chromatin compartment. Rad51 protein was also observed as 135 nm foci with no substructure and were also found to exhibit anti-correlation with 53BP1 foci, with the spatial relationship between the two proteins consistent between damage induced by high-LET alpha particles and low-LET protons (Reindl et al., 2017). To identify where particle damage is localized within the cell nucleus relative to chromosome position, Niimi et al. (2016). irradiated human fibroblasts with both X-ray and carbon ions, then located damage using fluorescence *in-situ* hybridization (FISH) and immunofluorescence to localize chromosome 1 and DSBs. As expected, damage from X-rays identified by γ-H2AX staining was evenly distributed throughout the chromatin versus carbon ions, where the damage accumulated at the boundary of chromosome 1 at greater than 4 times the frequency of X-ray induced damage.

Transmission electron microscopy (TEM) can achieve higher (∼20 nm) resolution (Winey et al., 2014), and, by gold particle labelling Ku70^S6p^ (pKu70) and DNA-PKcs^T2609p^ (pDNA-PKcs) after X-ray irradiation, has revealed that more clustered DSBs occur within the dense heterochromatin compared to euchromatin (Lorat et al., 2012). Even with exposure to low-LET radiation, persistent clusters of 2–3 DSBs (4–6 pKu70 foci) were observed, and it was postulated that these formed in heterochromatin and were not detectable until after large-scale chromatin relaxation. Another important repair factor found in high density within heterochromatin is 53BP1, observed in clusters of 30–60 labelled beads that did not colocalize with pDNA-PKcs, suggesting alternative repair pathways used to fix DSBs beyond NHEJ (Lorat et al., 2012). By comparison, using the same TEM approach to study the effects of carbon ion irradiation the same high density of damage and pronounced clustering was evident in the heterochromatic regions of the cell (Lorat et al., 2021). Damage within euchromatin mainly consisted of simple DSBs (pKu70 clusters with 1–2 beads within ≤10 nm), many of which were repaired after 5 h post-IR. In contrast, in the heterochromatin a higher proportion of DSBs were clustered (pKu70 clusters with 3–4, or ≥5 beads within ≤10 nm), and these clustered DSBs persisted out to 5 h post-IR, indicating delayed and potentially inefficient repair. These results add to the growing body of evidence that, for DSB induction in cellular DNA, not only does the quality of radiation impact the complexity with which DSBs are induced, but the chromatin complexity plays an important role as well.

## 3 High-LET IR Induced Damage Repair Signaling and Early Response

### 3.1 Association of DNA Repair Proteins With Clustered Damage

In assessing the presence and type of damage in a chromatinized context, more isolated damage from high-LET IR sources is detected in euchromatin, whereas clustered and complex damage tends to be more prevalent in heterochromatin (Asaithamby, Hu, and Chen 2011; Schwarz et al., 2019; Lorat et al., 2016, 2021). Widefield, confocal, and super-resolution microscopy have been used to identify colocalization of DNA damage response proteins and their accumulation at sites of clustered damage after high-LET IR exposure. Stimulated emission depletion microscopy has been used to monitor accumulation of 53BP1, Rad51, and BRCA1 after alpha particle irradiation (Schwarz et al., 2019), finding a persistence of both 53BP1 and BRCA1 in residual foci 24 h post IR, which appear larger than those initially induced, which has been previously observed for both 53BP1 and γ-H2AX. As expected, with increasingly complex lesions generated by exposure to alpha particles a large proportion (60%) of irradiated cells accumulated the HR factors BRCA1 and Rad51, which peak at 4 and 8 h post-IR, respectively. At these later time points, the repair of 53BP1 associated DSBs slows and over half the exposed cells contain 53BP1 foci after 24 h post-IR.

Imaging individual DNA repair proteins in relation to damage clusters has been applied extensively to differentiate induction and repair in euchromatin vs. heterochromatin. Beyond DSB markers such as γ-H2AX and 53BP1, XRCC1^(S518p, T519p, T523p)^ (pXRCC1) has been used to demarcate accumulation and repair of SSBs after exposure to low- and high-LET IR (Lorat et al., 2016). From this work, clusters composed of pKu70 and pXRCC1 were identified within heterochromatin 0.5 h after human fibroblasts were irradiated with carbon ions after initial exposure. Five hours post-IR, pXRCC1 signal in euchromatin disappeared, suggesting effective SSB repair, but persisted alongside pKu70 at clustered damage sites within heterochromatin (Lorat et al., 2016).

**FIGURE 4:**
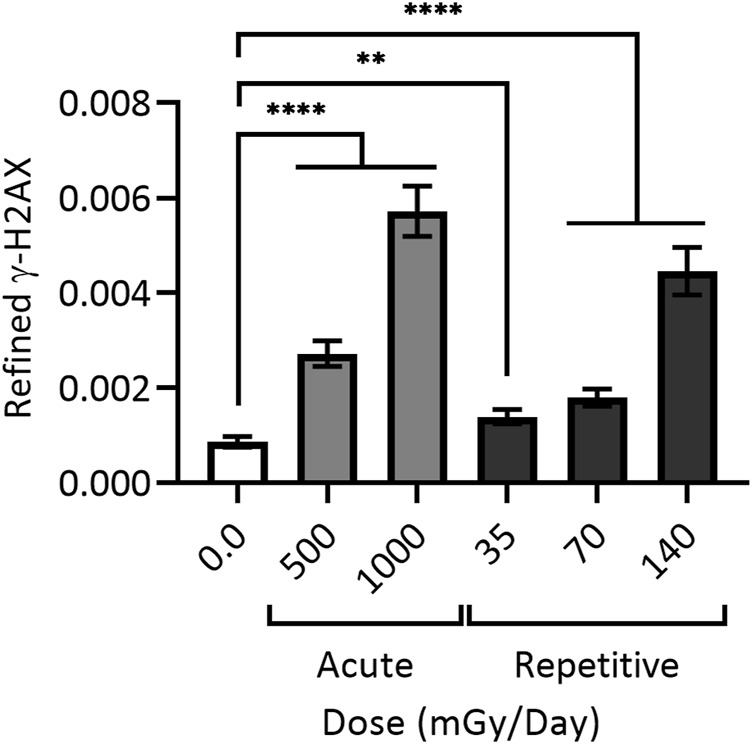
Cumulative DNA damage observable with (daily) repetitive alpha particle exposure even at low doses (<100 mGy). 48BR cells were given a one-time (acute) dose of 500 or 1,000 mGy particle IR (light grey) or repetitively exposed to 35, 70 or 140 mGy particle IR once per day for 15 days (dark grey), with sham irradiated control (white). Repetitively irradiated cells were harvested 24 h after the final dose, whilst acutely irradiated cells were harvested 1 h post IR. All cells were then fixed, stained and imaged together, generating refined γH2AX signal; black bars = mean ± SEM of *n* = 3 (1,700 cells total per condition). ** = statistically significant (<0.01); **** = statistically significant (<0.0001). Refined γ-H2AX represents the average intensity of γ-H2AX objects per individual cell as a function of the size of the nucleus measured as DAPI volume, and corrected for signal expansion of γ-H2AX during DSB repair. For a more detailed explanation of refined γ-H2AX, see (Stanley et al., 2020).

Work visualizing colocalization of 53BP1 with XRCC1 found similar results after exposure to 1 Gy of heavy silicon (Si) or iron (Fe) ions, as well as colocalization of 53BP1, XRCC1 and OGG1 (Asaithamby, Hu, and Chen 2011). To assess colocalization of all three proteins (and thus complex damage), cells were treated with 25 μM peroxide or 1 Gy gamma rays. For H_2_O_2_ treated cells, no colocalization was observed 0.5 h post treatment, whereas foci produced by gamma rays, heavy Si, or heavy Fe ions resulted in 4%, 40%, and 75% colocalization of all three markers, respectively; as expected, at 24 h post-IR, a subset of complex DSB damage sites persisted (Asaithamby, Hu, and Chen 2011). Research exploring the effects of low, repetitively delivered high-LET IR doses also documented a particular persistence of damage, with human primary skin fibroblast cells treated with 35–140 mGy alpha particle IR once every 24 h for 15 days leading to appreciable levels of persistent DSBs even at the lowest doses (Figure 4 **)** (Stanley et al., 2020).

In non-DSB clustered damage, artificially generated oligonucleotides with various combinations of base damage, including furans (stable surrogate AP-site), nucleotide gaps, and various forms of base damage including but not limited to 8-oxoG, thymine glycol, and 5-hydroxyuracil (5-hU), have been valuable tools in examining the processing of clustered lesions (Shikazono et al., 2009; Noguchi et al., 2012; Kozmin et al., 2021). Analyzing the mutational consequences of repair or misrepair of the oligonucleotides after transfection into yeast or mammalian cells has shown that opposing AP-sites (AP/AP) are readily converted into novel DSBs, where opposing 8-oxoG lesions were not (Malyarchuk et al., 2004; Kozmin et al., 2009). More complex combinations of 3–5 lesions in various configurations have revealed that combination of opposing AP-sites with an additional AP-site or 8-oxoG lesion on one of the strands can still produce DSBs, but at a slower rate (Eccles, Neill, and Lomax 2011). Inter-lesion spacing is an important factor in the generation of novel DSBs from the repair of non-DSB clustered damage, where distances of 3–8 nucleotides are sufficient to avoid interference of repair machinery at these clustered damage sites depending on the type of lesion present (Georgakilas et al., 2004; Shikazono et al., 2006; Kozmin et al., 2009). For example, it has been shown that no matter the inter-lesion distance, repair of two 8-oxoG lesions or an 8-oxoG and uracil on opposite strands will not lead to DSB formation in bacteria (Malyarchuk et al., 2004). More closely spaced or directly opposed base damage has been found to increase base mutation at these sites, and do not readily produce DSBs (Eccles, Neill, and Lomax 2011). Further details into combinations of base damage in non-DSB clustered DNA and the hierarchy of repair have been thoroughly reviewed in (Sage and Shikazono 2017).

### 3.2 Histone Post-translational Modifications and Chromatin Remodeling

In response to alpha particles or high-LET proton IR exposure, the ubiquitylation of histone H2B lysine 120 facilitated by the E3 ubiquitin ligases MSL2 and RNF20/RNF40 was induced several hours after irradiation (Carter et al., 2018). In contrast, an increase in the same H2B ubiquitylation mark was unobserved after exposure to low-LET sources of IR (low-LET protons, X-rays, and gamma rays). If these ubiquitin ligases were knocked down *via* siRNA, DNA damage persisted and cell survival decreased, suggesting the importance of ubiquitination of H2B for the processing and repair of complex damage induced by high-LET proton and alpha particle IR. This work is consistent with earlier findings that H2B monoubiquitylation by the RNF20/RNF40 complex is necessary for chromatin relaxation for the effective repair of DSBs in damaged cells (Moyal et al., 2011; Klement and Goodarzi 2014; Yu, Qin, and Lou 2020). Poly (ADP-ribosyl) ation (PAR) is a key SSB signaling molecule, produced by PAR Polymerase (PARP) enzymes using NAD + as a cofactor and releasing nicotinamide (Schreiber et al., 2002; Boehler et al., 2011). HPF1 changes the target specificity of PARP enzymes, promoting modification of histone serine residues (Gibbs-Seymour et al., 2016). For SSBR, PARP1/2 activity is critical for the timely repair of the strand breaks through its recruitment of XRCC1, in turn recruiting downstream repair factors (Caldecott 2003). PARylation is likely important in the repair of lesions induced by high-LET IR as PARP inhibitor treatment sensitizes cells (Césaire et al., 2019).

There is also a lack of knowledge in terms of how chromatin remodeling is controlled at sites of clustered and complex repair. We know from imaging break sites after high-LET IR damage induction that extensive remodeling is required, especially in heterochromatin, and there is a marked increase in damage site mobility, displaying a higher degree of movement within the nucleus for both low- and high-LET radiation, suggesting that small scale movements (over several μm) are characteristic of breaks (Kruhlak et al., 2006; Falk et al., 2008). In work utilizing live cell imaging of DSBs, a comparison of mobility between low LET X-rays and high-LET heavy nickel (Ni) ions, no substantial differences were observed between breaks, nor any significant number (<2%) of large scale (>5 μm) movements (Jakob et al., 2009). This observed small scale movement is thought to help promote homology search during HR (Jakob et al., 2009; Miné-Hattab and Chiolo 2020). For low-LET IR, this increase in mobility is mediated by Snf2 chromatin remodeling enzymes. In budding yeast, it has been demonstrated that the Ino80 remodeler is involved in the movement of difficult to repair DSBs to the nuclear periphery. A similar movement is observed in higher eukaryotes, where heterochromatic DSBs move to the periphery of heterochromatin domains for efficient repair and this process is mediated by F-actin nucleation and the activity of myosins (Ryu et al., 2015; Caridi et al., 2018; Lamm et al., 2020). Outside of DSB mobility, Snf2 remodelers are implicated in the repair of numerous lesions, but little work has been done specifically looking at the repair of DNA damage caused by high-LET IR. Some remodelers play important roles in the resolution of hard to repair lesions. The Ino80 remodeler aids in the repair of interstrand cross-links (Nagai et al., 2008; Horigome et al., 2014; Andreev et al., 2019). Similarly, the ISWI remodeler is required for the repair of Artemis-dependent DSB repair in heterochromatin (Klement et al., 2014). Despite these studies using low-LET IR, we can infer that these remodelers are important for the repair of clustered lesions given the overlap in types of lesions studied and those generated following high-LET IR. It is also almost certain that these are not the only remodelers that participate in the repair of clustered damage given the ever-growing list of remodelers involved in DNA repair. Identifying key remodelers required for repair of clustered lesions that are more efficiently induced following high-LET IR would be useful, given the developing interest in targeting chromatin remodelers in cancer therapy (Kaur, Daoud, and Eblen 2019).

## 4 Repair of High-LET IR-Induced Damage in the Context of Chromatin

### 4.1 Oxidative Lesions and SSBs After High-LET IR

**FIGURE 5:**
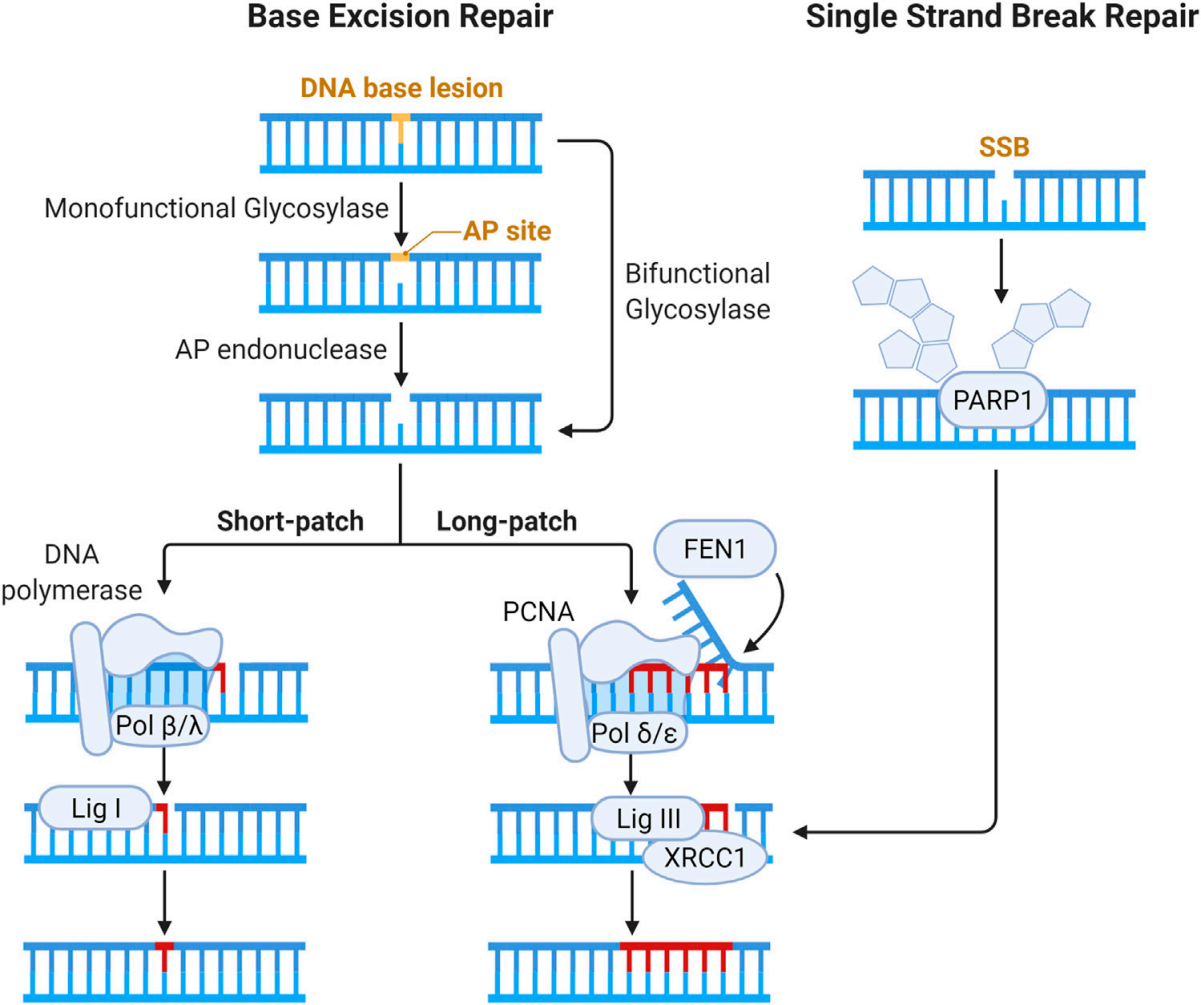
Schematic of pathways for base lesion and single strand break (SSB) repair. Base damage is recognized by a DNA glycosylase and subsequent enzymatic processing generates an abasic site. Abasic sites (also called AP, for apurinic/apyrimidinic sites) are processed by bifunctional DNA glycosylases or AP endonucleases, generating a gap in the DNA strand that can have a variety of ends requiring processing to enable ligation (Kim and Wilson III 2012). The gap is then filled by Polβ or Polλ via short patch BER, and the nick is resolved by XRCC1-DNA Lig III complex (Beard, Prasad, and Wilson 2006; Lebedeva et al., 2005; Cappelli et al., 1997). In long patch BER, Polδ and Polε fill in 2–12 nucleotides, forming an intermediary flap structure that is resolved by flap endonucleases (Matsumoto, Kim, and Bogenhagen 1994; Liu, Kao, and Bambara 2004; Wang, Wu, and Friedberg 1993). Flap removal generates SSB which is sealed by DNA ligase I. In direct SSB repair, damage is signaled by the activity of PARP1 (Masson et al., 1998). Gap filling and ligation in SSB repair are then accomplished in similar fashion to BER.

The SSB repair pathway is closely intertwined with base excision repair (BER) as, during BER, SSBs are generated as intermediates. The BER pathway recognizes and resolves base damage from oxidation, alkylation and deamination by specific DNA glycosylases. The steps of the BER and SSB pathways are illustrated below in Figure 5. The mechanisms involved in the repair of OCDLs have predominantly been ascertained through the use of site-specific induction of single base lesions in various combinations in plasmid DNA, which is then repaired with sets of purified proteins, removing this work from the context of chromatin and thus outside the scope of this review, and have been reviewed elsewhere (Blaisdell, Harrison, and Wallace 2001; Georgakilas 2008; Cadet et al., 2012).

### 4.2 Canonical DSB Repair Pathways After High-LET IR

**FIGURE 6:**
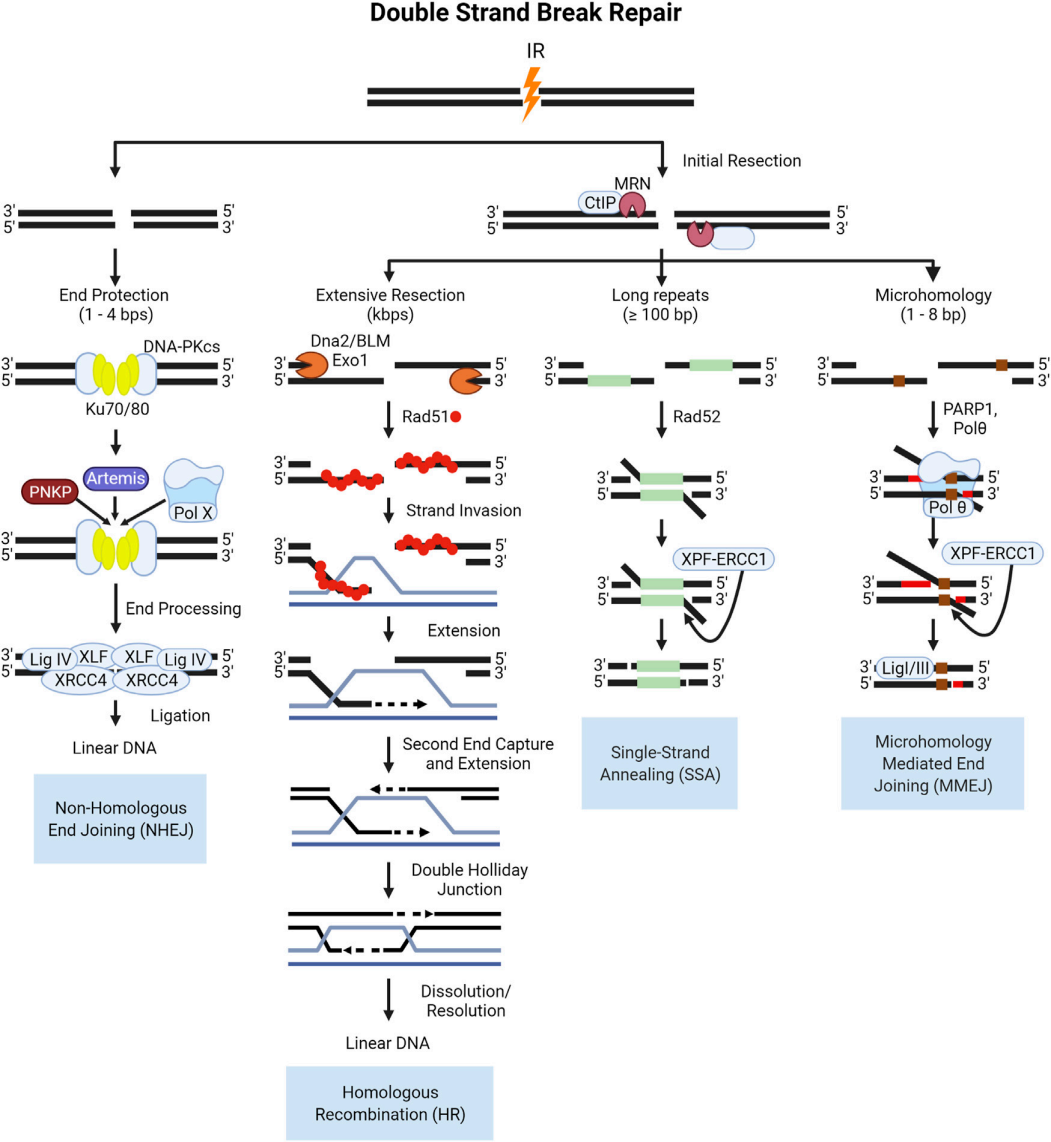
Schematic of end protected and resection dependent DNA double strand break (DSB) repair pathways. NHEJ is initiated by the binding of the Ku70/80 heterodimer which recruits DNA-PKcs, a kinase which functions to tether the break ends and recruit the factors involved in end processing and ligation shown here. In S- and G2, the increase in active cyclin dependent kinases (CDKs) in complex with cyclins phosphorylate key proteins like the resection mediating factor, CtIP which activates the nuclease activity of MRE11 in the MRN complex (Huertas et al., 2008; Makharashvili et al., 2014). The subsequent resection is the key event that commits a DSB to HR repair, producing single stranded 3′-DNA ends, which cannot be ligated by NHEJ (Williams et al., 2009; Himmels and Sartori 2016). Resected DNA is converted into a pre-synaptic filament with the replacement of RPA by the Rad51 recombinase protein, which then searches for homology (Thorslund et al., 2010; Ma et al., 2017; Sullivan and Bernstein 2018). Once homology is found, the filament invades the duplex DNA and the strand is elongated through synthesis (McVey et al., 2016; Wright, Shah, and Heyer 2018; Tavares et al., 2019). After the second end is captured and synthesized the resulting four strand structure, the double Holliday Junction, can then be dissolved or resolved (Svendsen and Harper 2010; Wyatt et al., 2013; Bizard and Hickson 2014; Wyatt and West 2014). In lieu of rad51 filament formation, in SSA Rad52 functions to anneal homologous DNA sequences and is always associated with the deletion of DNA between the two regions of homology. The resulting flap of DNA is subsequently removed by the XPF-ERCC1 nuclease complex after DNA ligation, which is enhanced by the presence of Rad52 (Motycka et al., 2004; Bhargava, Onyango, and Stark 2016). The occurrence of MMEJ has been correlated to the length of the 3′ overhang end, with longer ends of 45–100 bps being ideal for Polymerase θ (Polθ) helicase to act on the single stranded ends, facilitating the annealing of these ends as well as low fidelity polymerase activity for extension (Wyatt et al., 2016; Black et al., 2019). After extension, the 3′-flap overhangs are trimmed by the XPF-ERCC1 nuclease complex, and finally annealing is performed by Ligase I/III.

DSBs are repaired *via* two major pathways: non-homologous end joining (NHEJ) and homologous recombination (HR) repair (Goodarzi and Jeggo 2013; Vignard, Mirey, and Salles 2013; Liu, Olive, and Bristow 2008; Bhogal, Jalali, and Bristow 2009; Rothkamm et al., 2015). In all phases of the cell cycle, DSBs are repaired *via* NHEJ, which involves the direct ligation of DSB ends with some processing, resulting in more error-prone repair. The first proteins to accumulate at sites of DSBs are the sensor complexes of Ku70/Ku80 and MRE11-RAD50-NBS1 (MRN). The Ku complex is a critical component of NHEJ, recruiting the DNA-dependent protein kinase catalytic subunit (DNA-PKcs) to the break site which serves as a bridge to keep broken ends tethered. DNA-PKcs interacts with several end processing proteins, illustrated in Figure 6, to clean-up “dirty” ends, and once processed the ends are ligated, restoring the DNA. HR repair is an important pathway for cells undergoing replication or preparing for division in the synthesis (S-phase) and G2 phases of the cell cycle. Pathway choice between NHEJ and HR is an ever-evolving area of research due to the complexity of protein interactions and cellular conditions required for one pathway to function over the other. In the case of DSB repair of high-LET IR induced lesions, the literature supports the notion that NHEJ processes are fundamentally poor at, or possibly even “inhibitory” to, the resolution of highly clustered DSBs in a timely manner (Pang et al., 2011; Yajima et al., 2013; Takahashi et al., 2014). And whilst HR-mediated DSB repair is thought to be the primary pathway that resolves these lesions, this does not take place without introducing mutation (Shammas et al., 2009; Rodgers and McVey 2016).

### 4.3 Alternative DSB Repair Pathways After High-LET IR

Alternative pathways to repairing double strand breaks include single-strand annealing (SSA) and alt-EJ, which is sometimes referred to as microhomology mediated end joining (MMEJ) or pol-Theta mediated end joining (TMEJ). The canonical NHEJ pathway is the direct ligation of DNA ends and does not require complementary pairing, and can be performed with 1–4 nucleotide overhangs (Mladenov and George 2011; Seol, Shim, and Lee 2018). If there is extensive resection over several kilobases, HR is favored over the alternative pathways with the binding of RPA to the single stranded DNA acting as a negative regulator as it interferes with the annealing of complementary bases (Wang and Xu 2017; Liu and Kong 2021). In the absence of extensive resection, and with the presence of some degree of homology over several hundred base pairs, SSA proceeds as an alternative pathway for repair. The loss of DNA and potential for end migration leading to deletions or translocations makes SSA highly error prone. If there is micro-homology present between 2–20 bases on either side of a DSB and there is moderate resection catalyzed by the MRN complex and CtIP in mammalian cells, these regions may anneal. In biochemical studies, the depletion of long-range resection factors in yeast such as Sgs1 (mammalian BLM), and EXO1 led to a shift from HR repair to MMEJ for DSBs with microhomologies in the flanking sequences (Truong et al., 2013; Deng et al., 2014; Ahrabi et al., 2016). The steps of the canonical and alternative DSB pathways are illustrated in Figure 6.

### 4.4 Repair of Complex and Clustered DSBs

In this section, we discuss the repair pathway choice of high-LET induced DSBs that depends on the chromatin state in which the damage occurs, as well as the clustering and complexity of the damage. Pathway choice in euchromatin versus heterochromatin in mammalian cells is predominantly dictated by the accessibility of damage. DSBs induced by IR undergo biphasic repair kinetics, with fast and slow components influenced by the chromatin state [reviewed in (Jeggo, Geuting, and Löbrich 2011; Shibata and Jeggo 2020)]. The accessibility of euchromatin allows the accumulation of repair proteins and rapid repair of DSB damage. DSBs generated in euchromatin are primarily repaired by NHEJ, which is the major DSB repair pathway in human cells, as evidenced by a major DSB repair defect in both G1 and G2 after knockout of DNA Ligase IV, an essential NHEJ protein (Beucher et al., 2009). NHEJ accounts for the fast component of DSB repair, and contributes to the resolution of approximately 70%–80% of IR induced DSBs (Beucher et al., 2009; Shibata et al., 2011).

In heterochromatin, accessibility is limited and repair depends on the signaling of ATM kinase to recruit remodeling factors (MDC1, 53BP1) which relax the chromatin [reviewed in (Goodarzi, Jeggo, and Löbrich 2010)]. In some organisms, these breaks are relocated to the periphery of heterochromatin and contribute to the slow component of repair (Chiolo et al., 2011; Jakob et al., 2011; Neumaier et al., 2012)*.* There is evidence that breaks repaired as a part of the slow kinetics phase are predominantly resolved through resection mediated repair pathways in both G1 and G2 phases of the cell cycle (Shibata et al., 2011; Barton et al., 2014; Löbrich and Jeggo 2017). For damage induced by low-LET IR, the slow component accounts for approximately 20%–30% of repair, while for high-LET alpha particles the proportion is much higher, around 60%–80% (Antonelli et al., 2015; Stanley et al., 2020). For clustered damage, the ability of NHEJ to function normally is inhibited by the production of short DNA fragments. These fragments are bound by the Ku70/80 sensing proteins and the effector kinase DNA-PKcs, but DNA-PKcs kinase is inactive if bound to DNA fragments of ≤32 bp (Pang et al., 2011; Nickoloff, Sharma, and Taylor 2020). Additionally, using an I-*Sce*I engineered system to control the induction of multiple DSBs to simulate clustered damage, clustering of DSBs led to the suppression of canonical NHEJ as well as the increased formation of translocations relative to single DSB sites (Schipler et al., 2016).

In work aimed at assessing differences in DSB processing after exposure to high- and low-LET IR using live cell imaging, alpha particles induced fewer, but more intense DSB foci compared to an equivalent unit dose of X-rays, suggesting the production of closely spaced DSBs along the alpha particle trajectory (Roobol et al., 2020). It was also observed that the intensity of 53BP1 foci increased over time for X-ray induced DSBs, potentially indicating greater end protection, compared to the lack of equivalent increases in 53BP1 foci intensity in alpha particle treated cells (Roobol et al., 2020). Intensity can also be influenced by the longevity of the breaks, with signal intensity of chromatin markers increasing over time, which is observed for both low-LET photons as well as high-LET IR (Reindl et al., 2017; Schwarz et al., 2019; Roobol et al., 2020; Stanley et al., 2020). The presence of multiple RPA foci (marking resection) within single 53BP1 foci (marking a DSB) after alpha IR exposure suggests clustering of multiple resection events (Roobol et al., 2020). The accumulation of RPA at sites of damage after high-LET was also observed for heavy ion induced damage in G1 phase cells, in addition to the presence of resection mediating factors such as MRE11, CtIP, and EXO1 (Averbeck et al., 2014). These data support the paradigm of clustered or difficult to repair DSBs precluding canonical NHEJ as a mode of repair, and necessitating resection-based pathways (Beucher et al., 2009; Yajima et al., 2013; Biehs et al., 2017). Further evidence for the use of alternative, more error-prone pathways for the repair of particle IR-induced DNA damage is the induction of a greater number of structural chromosomal abnormalities, considered a hallmark of low fidelity repair by alt-EJ (Schipler et al., 2016; Mladenova et al., 2021). The consequences of misrepair and the presence of chromosomal aberrations after high-LET exposure will be discussed in the following section.

High-LET radiation also changes the condensation of chromatin along the particle trajectory (Timm et al., 2018). For simple DSBs generated in heterochromatin, the loss of ATM from resected DSBs reduces KAP-1^S824p^ (pKAP-1), and begins the restoration process of the heterochromatic super-structure (Geuting, Reul, and Löbrich 2013). It is proposed that the “re-heterochromatinization” of DNA after the initial resection step of HR prevents the initiation of alternative DSB repair pathways, primarily alternative end-joining (alt-EJ) (Geuting, Reul, and Löbrich 2013). In clustered damage induced with heavy ion radiation, TEM was used to monitor decondensed regions of chromatin along the particle track, and found small areas of decondensed chromatin around DSBs 5 h post-IR, which then partly re-condensed by 24 h and suggested a slow remodeling process after high-LET damage induction (Timm et al., 2018). In these experiments, pATM and pKAP-1 colocalized in heterochromatic regions adjacent to the decondensed areas along the particle damage track, as well as the presence of clustered (>2–3 pKu70 beads) DSBs (Timm et al., 2018). These data provide insight into the effects of clustered DSBs induced by high-LET IR on repair, in which canonical repair pathways are inhibited (NHEJ), as well as organizational effects when induced within heterochromatin, whereby recondensation of heterochromatin after resection of clustered DSBs is impeded.

An additional layer of complexity is the presence of “dirty” ends which usually consist of abasic sites or base damage on the DSB tails. This presents a challenge for many of the DSB pathways, for example NHEJ and MMEJ, whose Ligase IV or Ligase I/III complexes require “clean” ends. In NHEJ, several enzymes which can function in restoring the ligatability of ends include the Polynucleotide Kinase 3′-Phosphatase, which is recruited during NHEJ by XRCC4 and restores 5′ phosphate and 3′ hydroxyl ends (Chappell et al., 2002; Koch et al., 2004; Weinfeld et al., 2011). Another important enzyme for removing dirty ends is the Artemis endonuclease. After IR but not etoposide, Artemis is required for 10%–20% of NHEJ or HR-mediated repair events in G0, G1, and G2 (but not S) phase cells (Riballo et al., 2004; Deckbar et al., 2007; Löbrich and Jeggo 2007; Goodarzi et al., 2008; Beucher et al., 2009). Finally, complex chemical adducts at DSB ends can be removed by the nuclease activity of the MRN complex [reviewed in (Sasanuma et al., 2020)].

**FIGURE 7:**
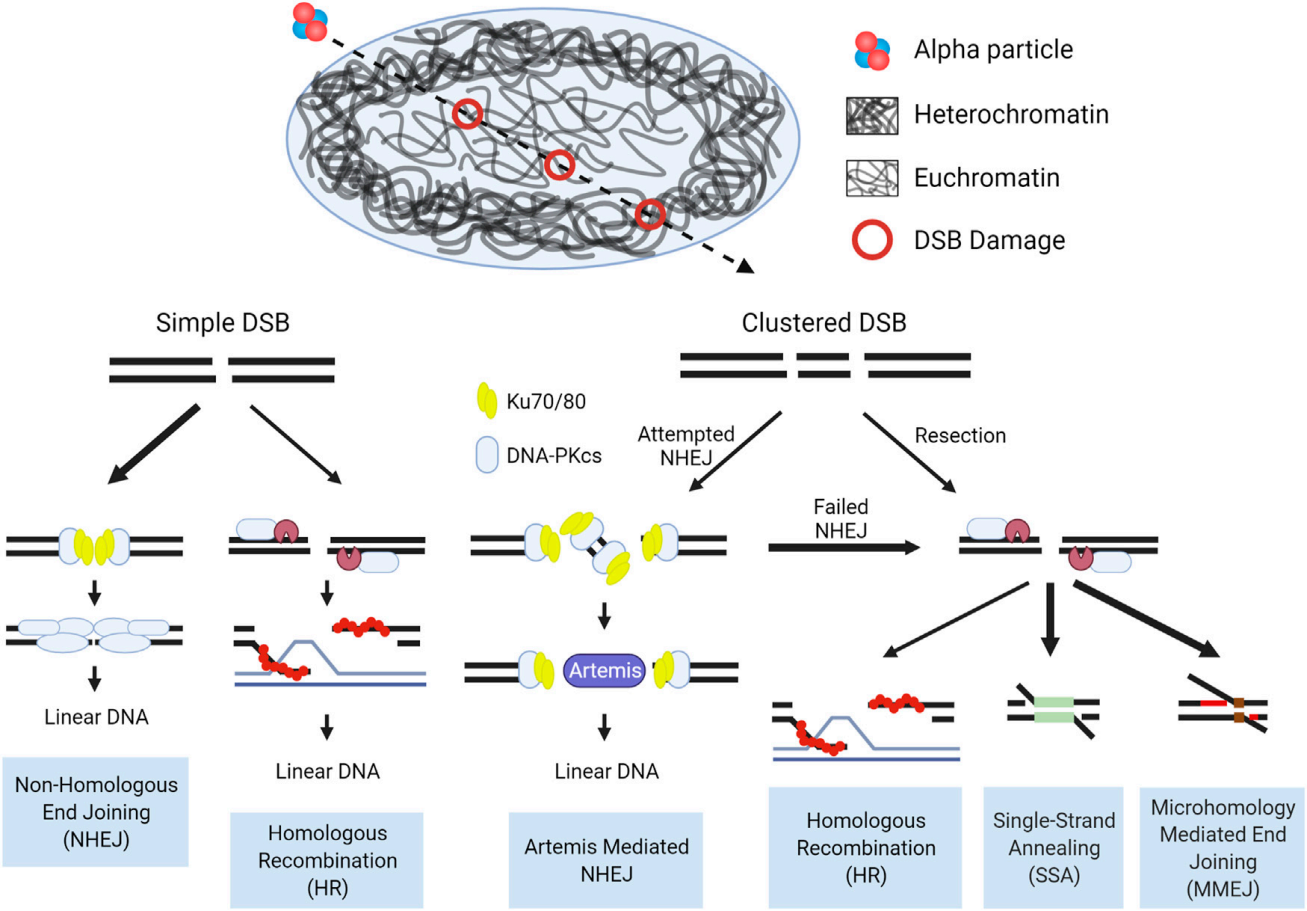
Schematic of DSB repair pathway choice for high-LET induced damage in the context of chromatin. In the repair of simple euchromatic DSBs NHEJ functions as the predominant repair pathway, with a portion of repair carried out by Artemis mediated NHEJ (G0/G1) or HR (S/G2). Initiation of NHEJ at sites of clustered damage is attempted, but due to the presence of short fragments which inhibit activation of DNA-PKcs, pathway choice shifts to resection mediated pathways. Hence, alternative, more error prone pathways are considered the major repair mechanism for high-LET induced clustered DSBs, but a more detailed mechanism for pathway choice in a chromatin context is still uncertain. Bolded arrows denote the ‘preferred’ pathway for repair. Detailed pathway schematics can be found in Figure 6.

Taken as a whole, the pathways involved in the repair of DSBs induced by high-LET IR are dictated by the accessibility of the lesions to damage repair machinery, and whether that machinery can adequately function once engaged with the damage. Chromatin state drives the accessibility, with damage induced in the open euchromatic regions repaired rapidly, predominantly by NHEJ. High-LET induced clustered damage poses a barrier to effective NHEJ, whereby the repair machinery assembles at these breaks, but is inhibited when bound to short DNA fragments. This in turn shifts repair towards resection dependent pathways. In the context of tightly packed heterochromatin, the density of DNA results in more complex and clustered damage and with accessibility drastically reduced, relaxation and migration are required to repair these lesions. The observable increases in structural chromosomal abnormalities after high-LET IR exposure implicate a shift in repair from HR to the more error prone alternative pathways. These concepts are illustrated in Figure 7.

## 5 Cell Fate and Molecular Outcomes After Exposure to High-LET IR

A key characteristic of clustered and complex damage induced by high-LET IR is the difficulty by which it is repaired, and this often leads to damage persistence. If repair fails, 53BP1 foci persists, and continues to accumulate and support the activation of ATM signaling, which promotes ongoing cell cycle arrest. At this point, a cell has three primary “options”: 1) it can continue to attempt repair of persisting lesions, albeit likely using error prone alternative DSB repair pathways; 2) the cell cycle arrest becomes permanent, and cells become senescent; or 3) constant damage signaling activates the apoptotic pathways and the cell dies. The induction of senescence and apoptosis is very often cell type dependent, as reviewed in (Childs et al., 2014; Faget, Ren, and Stewart 2019; Roger, Tomas, and Gire 2021). Additionally, the induction of apoptosis and senescence after exposure to IR can also depend on the innate radiosensitivity of the cell line based on its genetic background (Balcer-Kubiczek 2012; Mirzayans, Andrais, and Murray 2017).

### 5.1 Genome Stability and the Consequences of Misrepair

**FIGURE 8:**
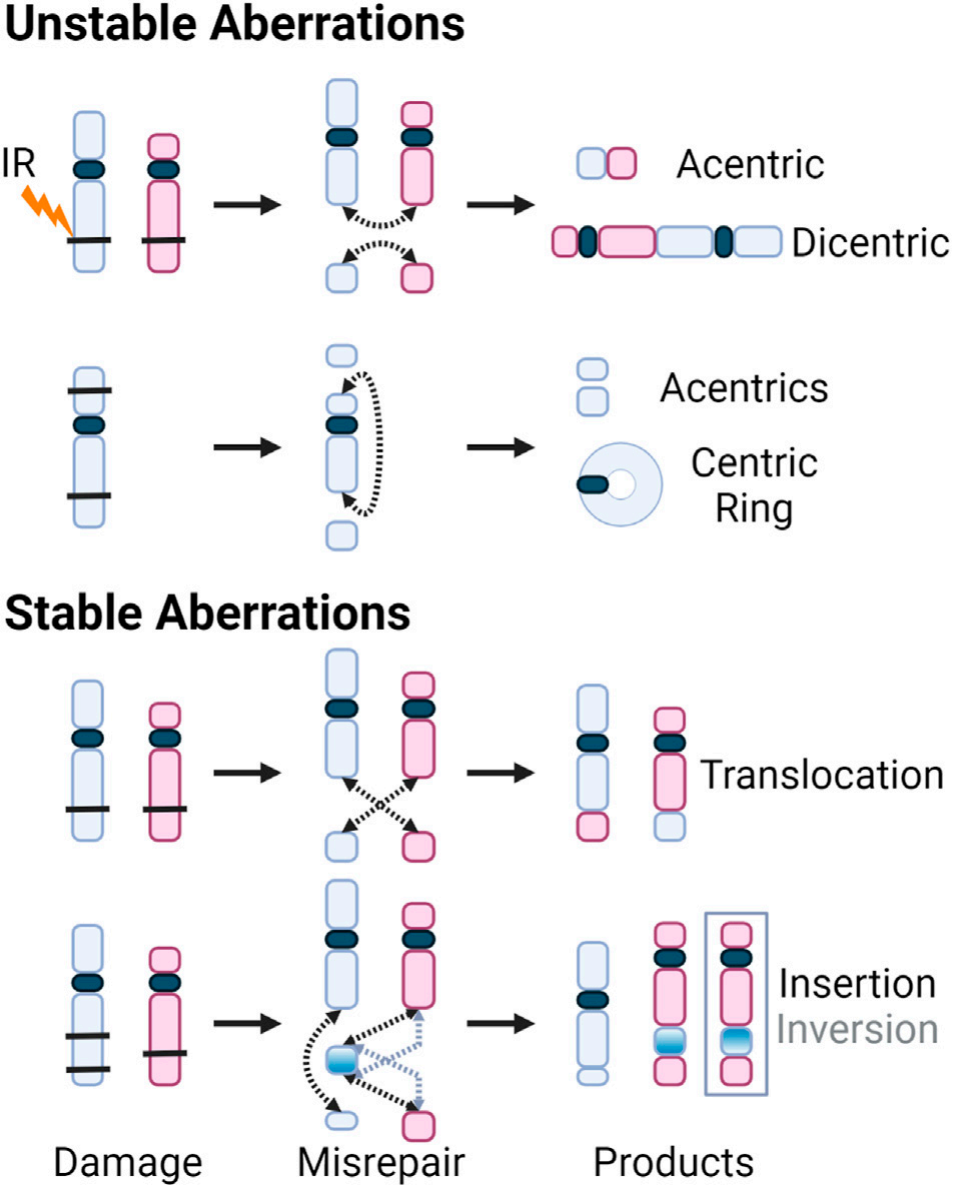
DSBs caused by exposure to IR (black bars) shown on chromosomes with centromeres (blue and red, and dark blue, respectively). Dotted arrows indicate where the breaks rejoin. Products of the various misrepair scenarios include unstable aberrations: acentric and dicentric chromosomes, centric rings; and stable aberrations: translocations, as well as insertions and inversions. For insertions and inversions, black arrows show where the fragments join during an insertion, and grey arrows show where the inverted chromosomal fragment joins. The inversion is further illustrated by inverting the color gradient of the inserted chromosome fragment and the final inversion product is enclosed in a grey box.

Although DSB repair through HR is considered error-free, all major pathways of damage response can result in erroneous repair due to the complexity of clustered damage after exposure to high-LET radiation. The misrepair of DSBs leads to chromosomal aberrations, with two main subcategories, those which are stable and can be passed on to daughter cells, and unstable aberrations which cannot. The transmissible stable aberrations, such as translocations, inversions or insertions, can persist in the dividing cell population and can lead to carcinogenesis (Aplan 2006; Burrow et al., 2009; Anderson 2019). Unstable aberrations like acentrics, dicentics, and centric rings, as the name suggests, are too unstable to survive cell division. Consequences of unstable chromosome aberrations include anaphase bridges and micronuclei, in which there is large-scale loss of genomic information and typically result in cell death (Kaddour et al., 2017). Anaphase bridges formed by dicentric chromosomes can break during cytokinesis, resulting in the loss of the telomere at the end of the chromosomes (Lo et al., 2002; Selvarajah et al., 2006). These partial chromosomes can fuse with other incomplete chromosomes, inducing “breakage-bridge-fusion” cycles, leading to prolonged instability (Gascoigne and Cheeseman 2013; Barra and Fachinetti 2018). The various aberrations are illustrated below in Figure 8.

Staining strategies such as FISH and multiplexed-FISH (M-FISH), have been used to assess chromosomal translocations after alpha particle exposure (Anderson et al., 2000; Anderson, Stevens, and Goodhead 2002; George et al., 2003; Tawn et al., 2007; Curwen et al., 2012). These lesions are considered stable and arise from the recombination and fusion of at least two different chromosomes after formation of at least two DSBs (Hakim et al., 2012; Herate and Sabatier 2019; McKenna et al., 2019). The study of alpha particle effects using FISH approaches have analyzed the presence of simple and complex exchanges, classified based on the minimum number of chromosomes, arms, and breaks (C/A/B) involved. In work from Anderson et.al., 2002 quantifying the damage induced by alpha particles passing through peripheral blood lymphocytes, the passage of a single high-LET alpha particle through a nucleus typically results in the production of a single complex exchange with average C and B values of 5.37 and 7.36, respectively. In research exploring the transmissibility of stable aberrations, on average misrepair of 3–5 breaks in 3–4 chromosomes was observed in naïve T-lymphocytes differentiated from surviving populations of alpha irradiated hematopoietic and progenitor cells 15 days after initial exposure to 0.5 Gy of alpha particles (Sumption, Goodhead, and Anderson 2015).

In a recent study, M-FISH was used to assess the effects of *ex vivo* exposure of peripheral blood lymphocytes to alpha particles from 0 to 2 Gy (Hartel et al., 2021). For this analysis an additional time point after damage induction was assessed 72 h post-IR. Although it is well known that high-LET IR can induce mitotic delay, previous studies using alpha particles have only examined early times (Ritter et al., 2002; Tawn et al., 2007). At 48 h, alpha particle IR induced a greater number of complex exchanges compared to X-ray IR, which are defined as the occurrence of ≥3 breaks across ≥2 chromosomes; this has been thoroughly reviewed in (Anderson 2019). By 72 h, there were greater numbers of aberrant cells, aberrations, breakpoints, and complex exchanges for alpha particle IR exposure vs. an equivalent dose of X-rays. The researchers also identified a greater relative number of unstable aberrations produced by alpha particles per dose, suggesting the complexity of damage leads to a greater degree of cell killing and non-proliferation compared to low-LET X-ray exposure, resulting in a lower risk of long-term heritable cytogenetic damage from alpha particle exposure (Hartel et al., 2021). The authors caution against extrapolating these results to other cell lines given the radiosensitivity of peripheral blood lymphocytes, as well as with heavy ions, which produce a much wider track and may result in different ratios of stable to unstable aberrations. Given these cautions, additional research is required and may benefit from more sensitive methods for assessing structural changes such as sequencing, as well as testing additional sources of high-LET IR in additional cell lines.

As discussed, generation of clustered damage occurs primarily in heterochromatin following irradiation by high-LET IR (Asaithamby, Hu, and Chen 2011; Lorat et al., 2012; Lorat et al., 2021). It has also been observed that cells with a portion of unrepaired clustered lesions induced by heavy ions are able to progress into mitosis, resulting in large numbers of chromosomal aberrations compared to low-LET IR (Asaithamby, Hu, and Chen 2011). Additionally, at the highest LET used for these experiments (heavy Fe ions, LET = 150 keV/μm) persistent lesions were found in both heterochromatic and euchromatic regions (Asaithamby, Hu, and Chen 2011). To date, much of the research assessing the aberrations generated in the aftermath of high-LET IR exposure has not sought to differentiate between the misrepair of lesions in euchromatin and heterochromatin, and additional work is necessary for assessing the proportion of aberrations induced in the context of chromatin.

As discussed in Section 4, the two major alternatives to canonical NHEJ and HR are alt-EJ/MMEJ and SSA. In mammalian cells, MMEJ repair only requires small 1–8 bp segments of microhomology in order to rejoin break ends, which typically results in small deletions (Pannunzio et al., 2014). However, because a key step in this pathway is DNA synthesis across gaps *via* polymerase θ, it can result in templated insertions (Yu and McVey 2010; Zahn, 2015; Carvajal-Garcia et al., 2020). By contrast, SSA results in larger deletions, due to the use of longer homologies >20 bp favored by longer resection (Bhargava, Onyango, and Stark 2016). In the context of simple DSBs, pathway choice has been extensively studied in the context of cell cycle, chromatin state, and alternative pathways (Shibata 2017; Noordermeer et al., 2018; Scully et al., 2019; Escribano-Díaz et al., 2013; Arnoult et al., 2017; Janssen et al., 2019; Ferrand, Plessier, and Polo 2021; Ceccaldi, Rondinelli, and D’Andrea 2016; Sallmyr and Tomkinson 2018); however, the proportion of damage repaired by these alternative pathways in the context of high-LET IR damage requires further study.

Consequences of misrepair may lead to large scale chromosome aberrations *via* translocations, insertions or deletions, or other patterns of mutations potentially culminating in a signature from the mutagenicity of repair. However, the generation of an abundance of irreparable breaks after high-LET exposure might lead to cancer through a chromothripsis-like mechanism. Chromothripsis is thought to be the shattering of a chromosome leading to extensive rearrangements (Koltsova et al., 2019). Therefore, the analysis of sequence data beyond cytogenetic approaches may reveal a complex pattern of mutational accumulation in tumor suppressor genes or other genomic sites. There has been some research exploring the potential mutational signature of alpha particles by way of radon exposure *in vivo*. By comparing sequence data from individuals with known radon exposure, and our current knowledge of mutation signatures compiled *via* the Catalogue Of Somatic Mutations In Cancer (COSMIC), an alpha particle specific signature may bear a resemblance to known signatures 2 and 6 (Alexandrov et al., 2013; Lim et al., 2019). For example, signature 2 exhibits mutational accumulation associated with overactivation of the cytidine deaminase family of proteins (ABOBEC) leading to a mutational signature associated with BER and replication (Alexandrov et al., 2013). Signature 6 shows an accumulation of substitutions and indels characteristic of microsatellite instability, suggesting defects in mismatch repair (MMR) (Boland and Goel 2010; Alexandrov et al., 2013). In the context of heavy ions, work done in mammary tumors induced by high-LET heavy Fe ions and low-LET gamma rays in p53 deficient mice found that heavy Fe ion induced tumors were characterized by an increase in focal structural variants, particularly increased insertions and deletions as well as mixed chromosomes, in comparison to an equivalent dose of low-LET gamma rays which exhibited more non-focal SVs like whole chromosome aneuploidy (Li et al., 2020). COSMIC mutational signatures extracted from tumors induced by both radiation types were signatures 18, 5, and 3, associated with ROS induced damage, a clock signature, and defective HR repair, respectively (Alexandrov et al., 2013). COSMIC signature 18 was significantly more prevalent in tumors induced by gamma rays (Li et al., 2020). Additional studies are needed to confirm these signatures and to better understand the mutagenic consequences of exposure to high-LET radiation. In terms of signature association with euchromatin vs. heterochromatin, the COSMIC single base substitution signature 8, which is common in cancer, is enriched in gene-poor, heterochromatic regions (Singh et al., 2020). The authors suggest that rapid proliferation during cancer progression leads to an accumulation of errors during the late stages of replication, concentrating in these heterochromatic regions as a possible etiology for this signature (Singh et al., 2020). Further to this, in cancers with defects in transcriptionally coupled nucleotide excision repair (TC-NER), there is a strand bias in accumulation of mutations contributing to a signature which are more commonly found in euchromatic regions (Jager et al., 2019). There has been some research into combining COSMIC signatures with high-throughput chromosome conformation capture (Hi-C), which was used to assess differences in mutational processes acting in transcriptionally active vs. non-active chromatin in follicular lymphoma and chronic lymphocytic leukemia (Sepulveda-Yanez et al., 2021). To the best of our knowledge, no such work has been performed for ionizing radiation exposure.

### 5.2 Senescence and Apoptosis Induced by Unrepaired DNA Damage After High-LET IR

The consequences of persistent DNA damage signaling include senescence or apoptosis, with high-LET IR causing relatively higher levels of both phenomena than low-LET IR per unit dose, in exposed cells. The persistence of DSBs results in continual damage signaling, which in turn can serve to trigger permanent cell cycle arrest (Pack, Daigh, and Meyer 2019; Kumari and Jat 2021). Specifically, unresolved DSBs continuously activate the ATM and ATR kinases, which phosphorylate key proteins in the cell cycle arrest pathways, such as p53 (Harris and Levine 2005; Hurley and Fred 2007; Fridlyanskaya, Alekseenko, and Nikolsky 2015). In this context, permanent exit from the cell cycle is referred to as stress-induced premature senescence (SIPS). In one study, after exposing bone marrow mesenchymal stromal cells with low-LET X-rays or high-LET alpha particles, a higher percentage of senescent cells was observed after alpha particle treatment at an equivalent IR dose (Alessio et al., 2017). Using a mouse model system and measuring the expression of p16 to gauge senescence, exposure to 1 Gy of low-LET protons or high-LET heavy Fe ions resulted in a significant increase in p16 positive bone marrow cells, compared to sham irradiated controls (Kumar et al., 2022). Additionally, the Fe ions induced higher levels of senescence than low-LET protons, which had roughly 40% and 20% p16 positive cells, respectively, compared to 12% of sham irradiated control mice (Kumar et al., 2022). The current literature thus suggests that senescence is dependent on radiation quality, with high-LET radiation being more efficient at establishing SIPS given the persistence of difficult to repair complex damage (Zhang et al., 2016; Alessio et al., 2017; Kumar et al., 2022).

Alternatively, in other cell types the persistence of DNA damage signaling has been linked to apoptosis (Markova, Belyaev, and Medicine 2011). As the basis for radiation therapy, the ability of radiation to kill cells has been extensively studied in cancer cell lines (Hartfiel et al., 2019; Sia et al., 2020; Fabbrizi and Parsons 2022). As might be expected, in comparative studies of gamma ray and carbon ions, consistent and significantly higher percentages of nuclear fragmentation and up to 2-fold higher caspase-3 activity were observed in carbon ion irradiated HeLa and HsiI cells versus gamma irradiated cells (Ghorai et al., 2014). Additional studies in cancer cell lines found varied levels of apoptosis between cell lines, but a consistent increase in apoptosis after exposure to high-LET sources versus a low-LET control (Bertrand et al., 2014; Carter et al., 2018; Hartfiel et al., 2019; Nickson et al., 2021). In a recently published review, attention was drawn to the extensive knowledge of the apoptotic mechanisms of low-LET IR exposure, while the precise mechanisms of high-LET IR induced apoptosis and other modes of cell death have not been thoroughly researched and remains an important avenue of future work (Fabbrizi and Parsons 2022).

## 6 Concluding Remarks

The importance of understanding the biological consequences of high-LET radiation is clear, given the scope of human IR exposure as a terrestrial environmental hazard, cosmic radiation, as well as in cancer treatment (Darby et al., 2005; Krewski et al., 2005; Cucinotta et al., 2006; Mohamad et al., 2017; Gaskin et al., 2018; Nelson, Andersson, and Wuest 2021; Pearson et al., 2021). In this review, we have summarized the current understanding of lesions generated by high-LET IR exposure, how the cell repairs them in a chromatinized context, and the consequences of persistent or misrepaired damage. A core aspect of high-LET radiation is the ability to generate clustered and complex lesions which, even at low doses, can cause difficulties (Stanley et al., 2020).

For clustered and complex DSBs, there are gaps in our knowledge in terms of pathway choice mechanisms governing repair via alternative DSB repair pathways (Zhao et al., 2020). There is also a need to perform cell fate measurements beyond the standard 24-h time frame (Schwarz et al., 2019). Taken together, there have been great strides made in the past decade in assessing the complexity of high-LET induced damage from alpha particles and heavy ions. Future work will be required to better characterize the role of chromatin remodelers during the process of clustered break repair, as well as the mechanisms underlying pathway choice involved in repairing these lesions and the resulting mutational patterns with which they are associated. Understanding how these lesions are repaired may allow for the identification of individuals in a population who may be at greater risk of cancer development arising from the mutational burden generated by environmental exposure to even low doses of radiation. Additionally, the interplay between remodelers and complex and clustered DNA damage repair could be leveraged into new therapeutic approaches, including combination chemotherapy targeting relevant chromatin remodelers and critical pathways in clustered damage repair to sensitize cancer cells to radiotherapy, specifically high-LET particle treatment.
